# PRISMA-Equity 2012 Extension: Reporting Guidelines for Systematic Reviews with a Focus on Health Equity

**DOI:** 10.1371/journal.pmed.1001333

**Published:** 2012-10-30

**Authors:** Vivian Welch, Mark Petticrew, Peter Tugwell, David Moher, Jennifer O'Neill, Elizabeth Waters, Howard White, the PRISMA-Equity Bellagio group

**Affiliations:** 1 Ottawa Hospital Research Institute, Ottawa, Canada; 2 London School of Hygiene & Tropical Medicine, London, United Kingdom; 3 Department of Medicine, University of Ottawa, Ottawa, Canada; 4 University of Ottawa, Institute of Population Health, Ottawa, Canada; 5 University of Melbourne, McCaughey Centre, Melbourne School of Population Health, Melbourne, Australia; 6 International Initiative for Impact Evaluation (3ie), Washington, D.C., United States of America

## Abstract

Vivian Welch and colleagues present consensus-based guidelines for reporting equity-focused systematic reviews, the PRISMA-Equity extension.

**Summary Points**

- There is a global imperative to tackle national and international health inequities— defined as unfair and avoidable differences in health.
- One step in reaching this goal is to improve the rigorous, scientific evidence base on the impacts of policies on inequities in health outcomes, resource allocation, and use.
- Systematic reviews are increasingly recognized as a valuable source of evidence for both health care and health systems decision making, yet very few systematic reviews report effects on health equity.
- We developed consensus-based reporting guidelines for equity-focused systematic reviews in order to help reviewers identify, extract, and synthesise evidence on equity in systematic reviews.
- Increased use of these reporting guidelines will help improve the reporting of effects on both inequities in health outcomes and health care use across gender, socioeconomic position, and other characteristics, both in systematic reviews and eventually primary research, thus contributing to the global agenda to improve health equity.

## Introduction

Health equity and social determinants of health remain high on international and national agendas. Recently, the report of the World Conference on Social Determinants of Health (October 2011) recognized the need for increased availability of data on inequities in health and resource allocation [1]. The Global Symposium on Health Systems Research in 2010 also considered equity to be of fundamental importance [2]. Despite such global commitment, there continues to be a dearth of evidence on the effects of policies on health equity [3].

Health equity is defined as the absence of avoidable and unfair inequalities in health [4]. The moral judgment of fairness involves an ethical debate about freedom, capabilities, and opportunities with consideration of context [5]. Rigorous scientific measurement and evaluation of the effects of policies on health equity is necessary to meet the goals of the World Health Organization Commission on Social Determinants of Health (WHO CSDH). Studies of the average effects of interventions, which control for confounding across individual and population-level characteristics, hide their impact on health equity. Using average effects to guide policy may even result in increases in health inequalities despite good intentions, as shown by an assessment of the impacts of country-level efforts in child health [6]. Well-designed, scientific evaluations that assess effects on health equity are needed to break the poverty trap [7].

Systematic reviews have, on one hand, been promoted as a useful and comprehensive source of evidence for decision making [8],[9], and, on the other hand, have been criticized by decision makers for not providing evidence about health equity [10],[11].

Systematic reviews can address health equity questions in one of three ways. First, they can assess effects of interventions targeted at a disadvantaged population as done in a review on school feeding for disadvantaged children [12]. Second, they can assess effects of interventions aimed at reducing social gradients such as the review on interventions to reduce the social gradient in smoking [10],[13]. Third, they can assess effects of interventions not aimed at reducing inequity but where it is important to understand the effects of the intervention on equity, such as lay health workers [14] or obesity prevention in children [15]. We estimate that approximately 20% of systematic reviews indexed in MEDLINE meet one or more of these criteria [16].

A focus on health equity in systematic reviews may uncover evidence on intervention-generated inequalities [17], lack of evidence and the need for further research [15], or greater absolute impact for the poorest due to their poorer health status. For example, vitamin A has the largest absolute impact on mortality reduction for children with lowest nutritional status [18]. However, few systematic reviews assess effects on health equity and those that do often provide insufficient detail to allow replication, including poor reporting of some population characteristics, subgroup analyses, and applicability judgments [19].

Reporting guidelines are designed to encourage completeness and transparency in reporting methods and results of systematic reviews, such as the Preferred Reporting Items for Systematic Reviews and Meta-Analyses (PRISMA) Statement. Whilst there is guidance on conducting equity-focused systematic reviews [20], there is no guidance on reporting them. This is important because several methodological issues are specific to reporting on systematic reviews with a major focus on equity, such as how disadvantaged populations are defined, how equity is incorporated into syntheses, and how to report on the applicability of review findings to disadvantaged populations or settings.

We therefore developed reporting guidelines for equity-focused systematic reviews, and had two main goals: (1) to provide structured guidance on transparently reporting these methods and results, and (2) to legitimize and emphasize the importance of reporting health equity results. We aim to contribute to improving the evidence base for evidence-informed, equity-oriented policy through wide dissemination of these reporting guidelines.

## Methods

**Figure 1:**
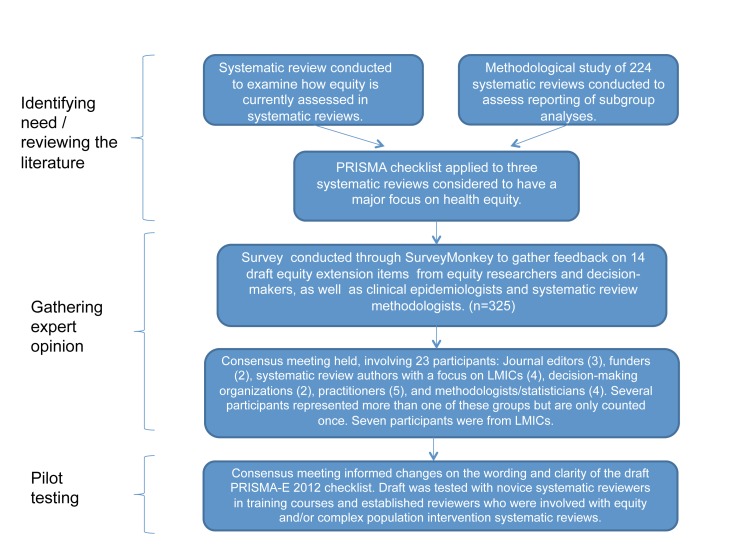
Flowchart of methods used to develop PRISMA-E 2012 reporting guidelines for equity-focused systematic reviews. LMIC, low- and middle-income country.

To produce these equity reporting guidelines (henceforth called PRISMA-E 2012), we followed recommendations [21] for the development of health research reporting guidelines: the relevant steps include identifying need, obtaining funding, reviewing the literature, conducting a broad survey (Figure S1; Tables S2, S3, S4, S5), and exploring consensus (see Figure 1).

### Consensus Meeting

A two-day consensus meeting was held on February 9–10, 2012 at the Rockefeller Foundation Sfondrata conference centre in Bellagio, Italy with 23 participants (Table S1). Its purpose was to discuss and reach consensus on each proposed item to be included in the PRISMA-E 2012 guidance. This guidance used the PRISMA statement and guidance as a starting point. PRISMA is an evidence-based minimum set of items for reporting in systematic reviews and meta-analyses; it does not currently have items relating explicitly to equity (www.prisma-statement.org) [22].

The meeting was organized to maximize participation and minimize lengthy presentations. Each participant was assigned a role to increase participation (Table S6). We provided all participants with instructions about these roles prior to the meeting (Table S7), and recorded all discussions and took detailed minutes.

We selected meeting participants on the basis of their expertise in systematic reviews, geographic diversity (prioritizing representation of participants from low- and middle-income countries), and representation of organizations that might use the reporting guidelines.

The participants included journal editors (*n* = 3), funders (*n* = 2), systematic review authors with a focus on low- and middle-income countries (*n* = 4), decision-making organizations (*n* = 2), practitioners in public health or health care (*n* = 5), and methodologists/statisticians (*n* = 4). Several participants represented more than one of these groups, but are only counted once. There were seven participants from low- and middle-income countries.

## Results

The consensus meeting participants (hereafter referred to as “we”) first agreed that the proposed equity reporting guidelines should be developed as a stand-alone document, because there is no set date for updating the PRISMA Statement and we felt that health equity issues are of sufficient global importance that they require explicit attention. Secondly, we agreed that the items were intended to focus on description of what was done and how, as with other reporting guidelines [21].

The proposed equity extension items are shown in Table 1, column 2, with the original PRISMA items in the left column. The rationale for these items is summarized below.

**Table 1:** Checklist of items for reporting equity-focused systematic reviews.

Section	Item	Standard PRISMA Item	Extension for Equity-Focused Reviews
**Title**			
**Title**	1	Identify the report as a systematic review, meta-analysis, or both.	Identify equity as a focus of the review, if relevant, using the term equity.
**Abstract**			
**Structured summary**	2	2. Provide a structured summary including, as applicable: background; objectives; data sources; study eligibility criteria, participants, and interventions; study appraisal and synthesis methods; results; limitations; conclusions and implications of key findings; systematic review registration number.	State research question(s) related to health equity.
	2A		Present results of health equity analyses (e.g., subgroup analyses or meta-regression).
	2B		Describe extent and limits of applicability to disadvantaged populations of interest.
**Introduction**			
**Rationale**	3	Describe the rationale for the review in the context of what is already known.	Describe assumptions about mechanism(s) by which the intervention is assumed to have an impact on health equity.
	3A		Provide the logic model/analytical framework, if done, to show the pathways through which the intervention is assumed to affect health equity and how it was developed.
**Objectives**	4	Provide an explicit statement of questions being addressed with reference to PICOS.	Describe how disadvantage was defined if used as criterion in the review (e.g., for selecting studies, conducting analyses, or judging applicability).
	4A		State the research questions being addressed with reference to health equity
**Methods**			
**Protocol and registration**	5	Indicate if a review protocol exists, if and where it can be accessed (e.g., web address), and, if available, provide registration information including registration number.	
**Eligibility criteria**	6	6. Specify study characteristics (e.g., PICOS, length of follow-up) and report characteristics (e.g., years considered, language, publication status) used as criteria for eligibility, giving rationale.	Describe the rationale for including particular study designs related to equity research questions.
	6A		Describe the rationale for including the outcomes (e.g., how these are relevant to reducing inequity).
**Information sources**	7	Describe all information sources (e.g., databases with dates of coverage, contact with study authors to identify additional studies) in the search and date last searched.	Describe information sources (e.g., health, non-health, and grey literature sources) that were searched that are of specific relevance to address the equity questions of the review.
**Search**	8	Present full electronic search strategy for at least one database, including any limits used, such that it could be repeated.	Describe the broad search strategy and terms used to address equity questions of the review.
**Study selection**	9	State the process for selecting studies (i.e., screening, eligibility, included in systematic review, and, if applicable, included in the meta-analysis).	
**Data collection process**	10	Describe method of data extraction from reports (e.g., piloted forms, independently, in duplicate) and any processes for obtaining and confirming data from investigators.	
**Data items**	11	List and define all variables for which data were sought (e.g., PICOS, funding sources) and any assumptions and simplifications made.	List and define data items related to equity, where such data were sought (e.g., using PROGRESS-Plus or other criteria, context).
**Risk of bias in individual studies**	12	Describe methods used for assessing risk of bias of individual studies (including specification of whether this was done at the study or outcome level), and how this information is to be used in any data synthesis.	
**Summary measures**	13	State the principal summary measures (e.g., risk ratio, difference in means).	
**Synthesis of results**	14	Describe the methods of handling data and combining results of studies, if done, including measures of consistency (e.g., *I* ^2^) for each meta-analysis.	Describe methods of synthesizing findings on health inequities (e.g., presenting both relative and absolute differences between groups).
**Risk of bias across studies**	15	15. Specify any assessment of risk of bias that may affect the cumulative evidence (e.g., publication bias, selective reporting within studies).	
**Additional analyses**	16	Describe methods of additional analyses (e.g., sensitivity or subgroup analyses, meta-regression), if done, indicating which were pre-specified.	Describe methods of additional synthesis approaches related to equity questions, if done, indicating which were pre-specified
**Results**			
**Study selection**	17	Give numbers of studies screened, assessed for eligibility, and included in the review, with reasons for exclusions at each stage, ideally with a flow diagram.	
**Study characteristics**	18	For each study, present characteristics for which data were extracted (e.g., study size, PICOS, follow-up period) and provide the citations.	Present the population characteristics that relate to the equity questions across the relevant PROGRESS-Plus or other factors of interest.
**Risk of bias within studies**	19	Present data on risk of bias of each study and, if available, any outcome level assessment (see item 12).	
**Results of individual studies**	20	For all outcomes considered (benefits or harms), present, for each study: (a) simple summary data for each intervention group; (b) effect estimates and confidence intervals, ideally with a forest plot.	
**Synthesis of results**	21	Present results of each meta-analysis done, including confidence intervals and measures of consistency.	Present the results of synthesizing findings on inequities (see 14).
**Risk of bias across studies**	22	Present results of any assessment of risk of bias across studies (see item 15).	
**Additional analysis**	23	Give results of additional analyses, if done (e.g., sensitivity or subgroup analyses, meta-regression [see item 16]).	Give the results of additional synthesis approaches related to equity objectives, if done, (see 16).
**Discussion**			
**Summary of evidence**	24	Summarize the main findings including the strength of evidence for each main outcome; consider their relevance to key groups (e.g., health care providers, users, and policy makers).	
**Limitations**	25	Discuss limitations at study and outcome level (e.g., risk of bias), and at review-level (e.g., incomplete retrieval of identified research, reporting bias).	
**Conclusions**	26	Provide a general interpretation of the results in the context of other evidence, and implications for future research.	Present extent and limits of applicability to disadvantaged populations of interest and describe the evidence and logic underlying those judgments.
	26A		Provide implications for research, practice, or policy related to equity where relevant (e.g., types of research needed to address unanswered questions).
**Funding**			
**Funding**	27	Describe sources of funding for the systematic review and other support (e.g., supply of data); role of funders for the systematic review.	

This checklist should be read in conjunction with the Statement and Explanation and Elaboration document, when available.

PICOS, participants, interventions, comparisons, outcomes, and study design.

**Title (1): Identify equity as a focus of the review, if relevant, using the term equity.** We agreed that it is very important to facilitate identification of reviews that address equity. We recognize this is not standard practice. In a search of all systematic reviews in the last year, we found only seven with equity in the title (Table S8). Thus, we propose that all equity-focused reviews include equity in the title.

**Structured Summary (2, 2A, 2B): State research question(s) and present results related to health equity, and assess applicability.** We recognize that end-users may only read the abstract. Therefore, it is critical that the abstract details effects on equity.

**Rationale (3 and 3A): Describe assumptions about mechanism(s) by which the intervention is assumed to have an impact on health equity, and provide analytic framework (if done).** We felt that equity-focused systematic reviews need to define a priori how the intervention is expected to influence health equity, so that these hypotheses can be tested. A visual representation (analytic framework) can be very useful. For example, they can show the causal chain [23]. Examples of using such frameworks for systematic reviews are increasingly available [24],[25].

**Objectives (4 and 4A): Describe how disadvantage was defined and research questions addressed.** Health equity and disadvantage are normative concepts, thus the systematic review must define how these terms are operationalized. Research questions related to equity need to be explicitly stated. For example, socially disadvantaged mothers were defined as those who were poor, lived in an inner-city environment, or were single parents in one review [26].

**Methods (6 and 6A): Provide rationale for eligible study designs and outcomes.** Equity questions may involve interventions and/or outcomes that have not been assessed through randomized evaluations. Therefore, we recommend explicit consideration of eligible study designs based on fitness for purpose [3],[20] and that the rationale for these choices be reported. Outcomes related to health equity need to be defined since they may involve differences between more and less advantaged groups. Furthermore, some questionnaire-based outcomes need to be adapted for less advantaged people. For example, a systematic review of culturally appropriate health education assessed the influence of culturally adapted measurement tools on knowledge outcomes using sensitivity analysis [27].

**Information sources and search strategy (7 and 8): Describe the relevance of databases and sources for equity as well as any terms used related to equity questions.** We recommend searching broadly for equity-related topics. Non-health databases such as transportation and economics may be relevant [28], as well as gray literature such as theses and unpublished reports. It is important to note that equity limits in the search could result in missing studies [29] unless these equity search strategies are validated. For example, the Cochrane Child Health filter [30] has been validated.

**Data items (11): List and define data items related to equity where such data were sought.** We recommend using an explicit checklist in the data extraction process to avoid missing data on health equity. We recommend place of residence, race/ethnicity/culture/language, occupation, gender/sex, religion, education, socioeconomic status, social capital and “plus” to indicate other possible factors such as disease status or disability (PROGRESS-Plus) [20]. Each of these characteristics requires careful consideration regarding their definition and classification as well as their interaction with other contextual elements and how they influence health inequities. For example, there is no agreed system for classifying race, ethnicity, and culture, particularly across different countries [31]. The concept of ethnicity has a relational dimension that helps in understanding social stratification and social exposures, and this relational dimension depends on context and setting [32]. The categorization of individuals according to ethnic groups may have different results when compared to individuals self-classification, and may also have negative consequences [31]. For each element of PROGRESS-Plus, authors need to consider whether health differences are avoidable and thus considered health inequities. When possible, systematic review authors should state how each element of PROGRESS-Plus was conceptualized, how it is hypothesized to affect health inequities, and how it was assessed. More limited criteria may also be used. For example, in a review of school feeding for disadvantaged children, data were sought on effects by socioeconomic position, or proxies for socioeconomic position such as nutritional status [12].

**Synthesis of results (14): Describe methods of synthesizing findings on health inequities.** In our view, an equity-focused review must present both relative and absolute differences between groups. Policy makers are interested in the absolute impact on deaths prevented or morbidity avoided. Furthermore, the absolute impact is likely to be higher in disadvantaged groups who are likely to have worse baseline health status. For example, vitamin A for preventing morbidity and mortality in children from 6 months to 5 years of age found a 24% relative risk reduction, which, in absolute terms, amounted to prevention of 22 deaths per 1,000 in a high-risk population but only three deaths per 1,000 in a medium-risk population [18].

**Additional analyses (16): Describe methods of additional synthesis approaches related to equity questions.** Additional quantitative or qualitative analyses may be necessary to answer equity questions; these include causal pathway analysis and process evaluation. For example, a review of school feeding tabulated effects for each study according to effect modifiers such as type of study, blinding versus unclear blinding, and high versus low energy [12].

**Results (18, 21, 23): Equity-focused reviews should report all relevant population characteristics as well as contextual factors that may be important for the population and intervention of interest.** We felt that the reporting guideline should emphasize the need to present results of all equity syntheses such as meta-regression and subgroup analyses (item 21), even if lacking in data. We also propose an additional item to describe the use of additional synthesis approaches such as process evaluation or the use of qualitative data to answer equity questions (item 23).

**Conclusions (26, 26A): Present extent and limits of applicability to disadvantaged populations of interest and implications for research, practice, or policy related to equity.** We agreed that systematic reviewers cannot make judgments about applicability or implications for practice and policy for every possible setting. However, we feel that the explicit reporting about applicability of findings to disadvantaged populations of interest is necessary to increase their relevance to decision makers (items 26 and 26A). For example, a Cochrane review of lay health workers suggested considerations for the assessment of applicability in different settings such as health system characteristics and on the ground constraints [33].

## Discussion

We have developed PRISMA-E 2012 to improve transparency and completeness of reporting of systematic reviews with a major focus on equity. We followed recent guidance on good practice in guideline development [21]. This inclusive and evidence-based process resulted in the proposal of 20 additional items to the PRISMA Statement to improve reporting of equity-focused systematic reviews.

Equity and fairness are of paramount importance to health care decision making and resource allocation [34]. This equity extension of PRISMA emphasizes the importance of enhancing the reporting of methods and results relevant to health equity, which we believe will improve the evidence base for equity-focused decision making over time.

We have planned several post-publication activities to increase dissemination and uptake of PRISMA-E 2012, such as presentations at conferences to reach decision makers, journal editors, and researchers, as well as web-based strategies such as webinars, an open-access website, and twitter. Readers can find out about post-publication activities on the Campbell and Cochrane Equity Methods Group website www.equity.cochrane.org.

To encourage journal endorsement, we will write to the nearly 200 PRISMA-endorsing journals and ask them to consider endorsing PRISMA-E 2012, using affirmative, precise language that has been shown to increase uptake, such as “[this journal] requires a completed PRISMA-E 2012 checklist as a condition of submission of systematic reviews whose main focus is equity. We recommend you, while completing this form, consider amending your manuscript to ensure your article addresses all issues raised by the PRISMA-E 2012 checklist, where appropriate. Taking the time to ensure your manuscript meets these basic reporting needs will greatly improve your manuscript, potentially enhancing its chances for eventual publication.” Similarly, we will contact funding agencies and those commissioning systematic reviews to consider recommending the use of PRISMA-E 2012. Grant peer review committees can also use the reporting guideline. We will evaluate the uptake of PRISMA-E 2012 by assessing the number of journals that cite PRISMA-E 2012 in their instructions for authors. We also plan to evaluate the influence of journal (and potentially other organizations) endorsement of the PRISMA-E 2012 by comparing endorsing and non-endorsing journals regarding reporting of PRISMA-E 2012 items in equity-focused systematic reviews [35].

Our development process has a number of strengths including the use of an inclusive process with input from intended users using a web-based survey and consensus meeting. The main limitations of our approach are that some terms are not widely accepted such as “logic model” and “analytic framework.” There is no universally accepted definition of “health equity.” We chose to use Whitehead's widely used definition as it differentiates simple differences in outcomes from those that are inequitable due to unfairness [4]. We acknowledge that our tool is limited to improving the reporting of reviews already examining equity, rather than improving equity research or indeed health equity itself.

In conclusion, we hope that the use of this PRISMA-E 2012 guidance will improve both the reporting and conduct of equity-focused reviews. In time, it may indirectly influence primary research by identifying the need for new research studies to answer health equity questions. Thus, we hope for this guidance to help contribute to the goals of the Rio Political Declaration on Social Determinants of Health [1] by improving the evidence base about equity in health outcomes, access, and use of health care.

## Supporting Information

Figure S1 **Survey on SurveyMonkey.**
(DOCX)

Table S1 **PRISMA-Equity Bellagio Group.**
(DOCX)

Table S2 **Equity extension of PRISMA.**
(PDF)

Table S3 **Distribution of online survey.**
(DOCX)

Table S4 **Characteristics of respondents of the survey (** ***n*** **= 423).**
(DOCX)

Table S5 **Responses to online survey.**
(DOCX)

Table S6 **Consensus meeting agenda.**
(DOCX)

Table S7 **Participant roles.**
(DOCX)

Table S8 **Systematic reviews with equity in the title (1-08-2011 to 1-08-2012).**
(DOCX)

## Abbreviations

PRISMA: preferred reporting items for systematic reviews and meta-analyses
PROGRESS-Plus: place of residence, race/ethnicity/culture/language, occupation, gender/sex, religion, education, socioeconomic status, social capital, and other possible factors such as disease status or disability
