# Supplementary figures and images for: PRISMA-Equity 2012 Extension: Reporting Guidelines for Systematic Reviews with a Focus on Health Equity
Source: PLoS Med. 2012 Oct 30;9(10):e1001333. doi: 10.1371/journal.pmed.1001333 (PMC3484052; doi:10.1371/journal.pmed.1001333)

**Figure S1: Survey on SurveyMonkey**


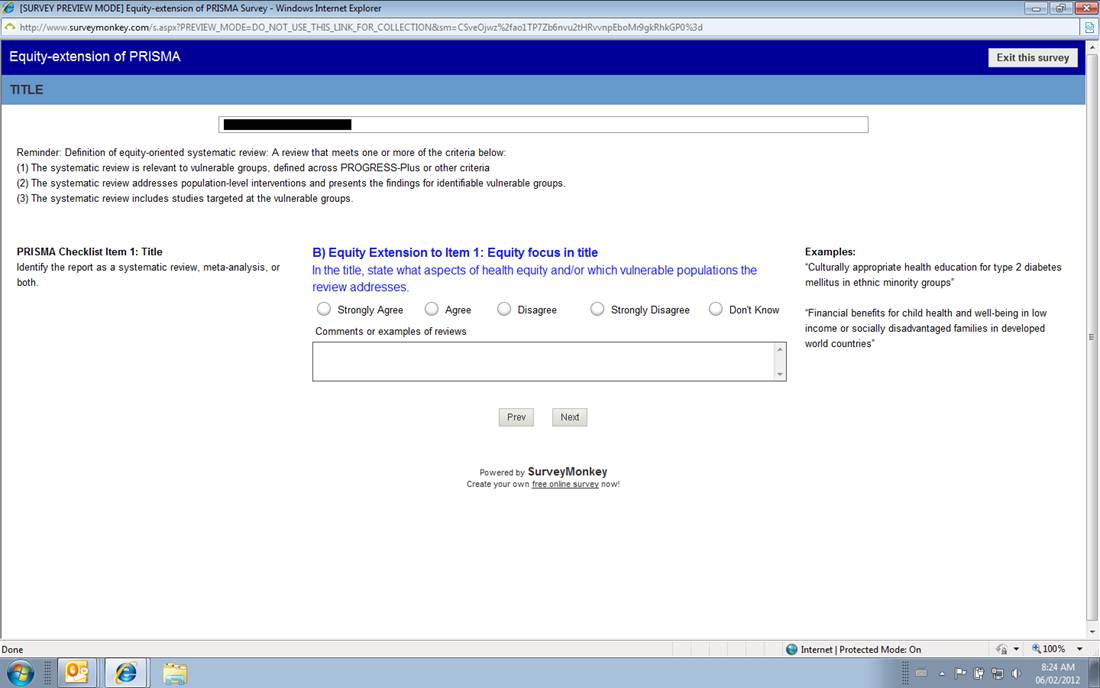

Supplement: Figure S1 — Survey on SurveyMonkey. (DOCX) [file pmed.1001333.s001.docx]
