# Supplementary material for: PRISMA-Equity 2012 Extension: Reporting Guidelines for Systematic Reviews with a Focus on Health Equity
Source: PLoS Med. 2012 Oct 30;9(10):e1001333. doi: 10.1371/journal.pmed.1001333 (PMC3484052; doi:10.1371/journal.pmed.1001333)
Supplement: Table S1 — PRISMA-Equity Bellagio Group. (DOCX) [file pmed.1001333.s002.docx]

**Webtable S1: PRISMA-Equity Bellagio Group**

| **Participant** | **Affiliation** | **Country** |
| --- | --- | --- |
| Rifat Atun | Imperial College London | UK |
| Shally Awasthi | King George’s Medical College | India |
| Virginia Barbour | PLoS Medicine | UK |
| Zulfiqar Bhutta | Husein Laljee Dewraj Professor and Chairman, Department of Paediatrics and Child Health, Aga Khan University Medical Center | Pakistan |
| Luis Gabriel Cuervo | Research Promotion & Development, Pan American Health Organization | USA |
| Jeremy Grimshaw | Ottawa Hospital Research Institute | Canada |
| Trish Groves | Deputy Editor, BMJ | UK |
| Tracey Koehlmoos-Perez | Health Systems and Infectious Disease Division, International Centre for Diarrhoeal Disease Research (ICDDR,B) | Bangladesh |
| Elizabeth Kristjansson | School of Psychology, Faculty of Social Sciences, University of Ottawa | Canada |
| David Moher | Ottawa Hospital Research Institute | Canada |
| Jennifer O’Neill | Campbell and Cochrane Equity Methods Group, Centre for Global Health, University of Ottawa | Canada |
| Andy Oxman | Global Health Unit, Norwegian Knowledge Centre for Health Services, Cochrane EPOC Review Group, WHO Advisory Committee on Health Research | Norway |
| Tomas Pantoja | Universidad Católica de Chile, Santiago, Systematic Review Methods Centre Cochrane EPOC editor, Alliance for Health Systems and Policy Research | Chile |
| Mark Petticrew | London School of Hygiene and Tropical Medicine, Campbell and Cochrane Equity methods group | UK |
| Terri Pigott | Campbell Methods Coordinating Group; Campbell Statistics Group, Loyola University Chicago | USA |
| Kent Ranson | Alliance for Health Systems and Policy Research, WHO | Switzerland |
| Tessa Tan Torres | World Health Organization, Choosing Interventions that are Cost-Effective (WHO-CHOICE) | Switzerland |
| Prathap Tharyan | Cochrane Evidence Aid, Department of Psychiatry; Cochrane Schizophrenia Group; Coordinator, South Asian Cochrane Network - India | India |
| David Tovey | The Cochrane Library | UK |
| Peter Tugwell | University of Ottawa, Cochrane Musculoskeletal Review Group, Campbell and Cochrane Equity methods group | Canada |
| Jimmy Volmink | Cochrane HIV/AIDS Review Group; South Africa Cochrane Centre, Medical Research Council of South Africa; Stellenbosch University, | South Africa |
| Liz Wager | Sideview | UK |
| Elizabeth Waters | Melbourne School of Population Health, The University of Melbourne | Australia |
| Vivian Welch | Ottawa Hospital Research, Campbell and Cochrane Equity Methods Group, Institute, Centre for Global Health, University of Ottawa | Canada |
| George Wells | Cardiovascular Research Methods Centre, University of Ottawa Heart Institute, Cochrane Non-Randomized Studies Methods Group |  |
| Howard White | International Initiative for Impact Evaluation (3ie); Campbell International Development Coordinating Group | India |
