# Supplementary material for: PRISMA-Equity 2012 Extension: Reporting Guidelines for Systematic Reviews with a Focus on Health Equity
Source: PLoS Med. 2012 Oct 30;9(10):e1001333. doi: 10.1371/journal.pmed.1001333 (PMC3484052; doi:10.1371/journal.pmed.1001333)
Supplement: Table S2 — Equity extension of PRISMA. (PDF) [file pmed.1001333.s003.pdf]

Survey Equity-extension of PRISMA – Proposed additional items or extensions of existing items for equity-oriented systematic reviews

We endorse the reasons behind PRISMA which was developed to improve the reporting of systematic reviews and meta-analyses. Systematic reviews have been called for to compile the evidence on how to reduce health inequities. Health inequities are differences in health which are both avoidable and considered unfair or unjust. Average effects may hide important differences, between the mean and the effectiveness of interventions in the poor and other disadvantaged groups so practitioners, policy makers and the public area need to be able to assess both.

For more information, please check our blogs at Speaking of Medicine (PLOS) and BMJ.

The survey will remain open until January 5th, 2012.

Definition of equity-oriented systematic review: A review that meets one or more of the criteria below:

1. The systematic review is relevant to vulnerable groups, defined across one or more of PROGRESS-Plus* or other criteria

Example: *Lay health workers in primary and community health care for maternal and child health and the management of infectious diseases* "More recently, the growing focus on the human resource crisis in health care in many LMICs has re-energised debates regarding the roles that LHWs may play in extending services to 'hard to reach' groups and areas; and in substituting for health professionals for a range of tasks."

2. The systematic review addresses population-level interventions and presents the findings for identifiable vulnerable groups.

Example: *Home safety education and provision of safety equipment for injury prevention.* "For objective (ii) studies were required to either report data on socio-economic characteristics or have unpublished data on socioeconomic characteristics available for the review. The socio-economic and demographic characteristics of interest were those previously found to be associated with an increased risk of childhood injury. These included child age, gender, ethnic group, family type (single or two parent), housing tenure, parental unemployment, residence in a deprived area, income, receipt of means-tested benefits, maternal education, maternal age, family size and overcrowding."

3. The systematic review includes studies that do not look at the whole population but are targeted at the vulnerable groups*

Example: *School feeding for improving the physical and psychosocial health of disadvantaged students* "Those studies in which children were classified as 'predominantly disadvantaged' by one or more of the following criteria: 1) Living in a rural area or village; 2) Living in an urban area and described as socio-economically disadvantaged (e.g. poor or low-income) or from poor areas (e.g. slums); 3) if statistics were presented showing that 30%of more of the children in the sample were underweight, or stunted (nutritionist judgement) or that the average weight, height, and Body Mass Index (BMI) were low (nutritionist judgement) and 4) studies were implicitly or explicitly aimed at disadvantaged children, and indicators of disadvantage were provided in the paper."

Health inequities are differences in health which are both avoidable and considered unfair or unjust. PROGRESS-Plus is one acronym to define factors across which inequities may exist. Other criteria are also used.

Equity-extension of PRISMA

***PROGRESS-Plus:** PROGRESS is an acronym for:

- Place of residence (rural/urban/inner city, low- or middle-income country)
- Race/ethnicity/culture
- Occupation
- Gender/sex
- Religion
- Education (literacy)
- Socioeconomic status
- Social capita

“Plus” refers to other categories across which discrimination may exist such as sexual orientation, age, disability, or disease status. Other lists of factors or criteria may be also used.

Instructions for Completing the 17 Question Survey

Please keep in mind as you answer questions:

1. We seek your opinion on these items as potential guidance for reporting equity-oriented systematic reviews.
2. These reviews may include randomized trials or non-randomized studies.
3. Please keep in mind that the survey items focus on guidance for reporting and not standards for the research methods.
4. You are encouraged to use the full range of the 5 point-scale. Only use the "Don't Know" option if you're really not sure.
5. Each item has a comments field if you wish to clarify your ratings or make suggestions for wording

PRISMA Checklist Item

The items from the PRISMA Checklist are listed in the right-hand column.

To view the complete PRISMA checklist click here.

Equity Extension to PRISMA Item

The proposed item for the equity extension is given in the centre column. Please use the five point scale to indicate whether you agree or disagree with the item. You can leave comments for each proposed item.

Example

An example is provided to give clarification for each proposed item for the equity-extension of PRISMA.

No relevant PRISMA item.

A) Reporting health equity is important in systematic reviews.

○ Strongly Agree ○ Agree ○ Disagree ○ Strongly Disagree ○ Don't Know

Comments

No example

TITLE

Equity-extension of PRISMA

Reminder: Definition of equity-oriented systematic review: A review that meets one or more of the criteria below:
(1) The systematic review is relevant to vulnerable groups, defined across PROGRESS-Plus or other criteria
(2) The systematic review addresses population-level interventions and presents the findings for identifiable vulnerable groups.
(3) The systematic review includes studies targeted at the vulnerable groups.

PRISMA Checklist Item 1: Title
Identify the report as a systematic review, meta-analysis, or both.

B) Equity Extension to Item 1: Equity focus in title
In the title, state what aspects of health equity and/or which vulnerable populations the review addresses.

○ Strongly Agree ○ Agree ○ Disagree ○ Strongly Disagree ○ Don't Know

Comments or examples of reviews

Examples:
"Culturally appropriate health education for type 2 diabetes mellitus in ethnic minority groups"

"Financial benefits for child health and well-being in low income or socially disadvantaged families in developed world countries"

ABSTRACT

Reminder: Definition of equity-oriented systematic review: A review that meets one or more of the criteria below:
(1) The systematic review is relevant to vulnerable groups, defined across PROGRESS-Plus or other criteria
(2) The systematic review addresses population-level interventions and presents the findings for identifiable vulnerable groups.
(3) The systematic review includes studies targeted at the vulnerable groups.

PRISMA Checklist Item 2: Structured Summary
Provide a structured summary including, as applicable: background; objectives; data sources; study eligibility criteria, participants, and interventions; study appraisal and synthesis methods; results; limitations; conclusions and implications of key findings; systematic review registration number.

C) Equity Extension to item 2: Assessment of equity
In the abstract, state whether and how health equity was assessed.

○ Strongly Agree ○ Agree ○ Disagree ○ Strongly Disagree ○ Don't Know

Comments or examples of reviews

Example: *Home safety education and provision of safety equipment for injury prevention* "We evaluated the effectiveness of home safety education, with or without the provision of low cost, discounted or free equipment in increasing home safety practices or reducing child injury rates and whether the effect varied by social group."

ABSTRACT

Equity-extension of PRISMA

Reminder: Definition of equity-oriented systematic review: A review that meets one or more of the criteria below:
(1) The systematic review is relevant to vulnerable groups, defined across PROGRESS-Plus or other criteria
(2) The systematic review addresses population-level interventions and presents the findings for identifiable vulnerable groups.
(3) The systematic review includes studies targeted at the vulnerable groups.

PRISMA Checklist Item 2: Structured Summary
Provide a structured summary including, as applicable: background; objectives; data sources; study eligibility criteria, participants, and interventions; study appraisal and synthesis methods; results; limitations; conclusions and implications of key findings; systematic review registration number.

D) Equity Extension to item 2: Effects on equity
In the abstract, state effects on health equity (e.g. the effects of the intervention on different subpopulations).

○ Strongly Agree ○ Agree ○ Disagree ○ Strongly Disagree ○ Don't Know

Comments or examples of reviews

Example: *Population tobacco control interventions and their effects on social inequalities in smoking: systematic review* “No strong evidence of differential effects was found for smoking restrictions in workplaces and public places, although those in higher occupational groups may be more likely to change their attitudes or behaviour. Smoking restrictions in schools may be more effective in girls. Restrictions on sales to minors may be more effective in girls and younger children. Increasing the price of tobacco products may be more effective in reducing smoking among lower-income adults and those in manual occupations, although there was also some evidence to suggest that adults with higher levels of education may be more price-sensitive. Young people aged under 25 are also affected by price increases, with some evidence that boys and non-white young people may be more sensitive to price.”

Reminder: Definition of equity-oriented systematic review: A review that meets one or more of the criteria below:
(1) The systematic review is relevant to vulnerable groups, defined across PROGRESS-Plus or other criteria
(2) The systematic review addresses population-level interventions and presents the findings for identifiable vulnerable groups.
(3) The systematic review includes studies targeted at the vulnerable groups.

PRISMA Checklist Item 3: Rationale
Describe the rationale for the review in the context of what is already known.

E) Equity Extension to item 3: Logic Model
In the background, provide or refer to a logic model/analytical framework to represent the pathways through which the intervention is expected to affect health equity.

○ Strongly Agree ○ Agree ○ Disagree ○ Strongly Disagree ○ Don't Know

Comments or examples of reviews

Equity-extension of PRISMA

Example: ***[The Effectiveness of Early Childhood Development Programs: A Systematic Review](#)*** **"The analytic framework used for the early childhood development program reviews, shown in Figure 1 [see below], is derived from the social environment and health logic model. In the logic model, "opportunities for education and for developing capacity" serve as intermediate indicators along a pathway linking resources in the social environment to health outcomes."**

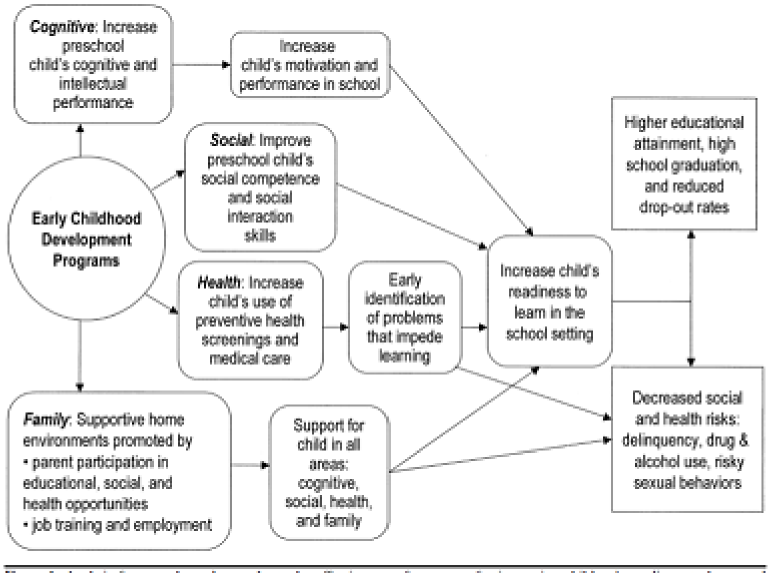

Figure 1. Analytic framework used to evaluate the effectiveness of programs for improving children's readiness to learn and preventing developmental delay.

INTRODUCTION

Reminder: Definition of equity-oriented systematic review: A review that meets one or more of the criteria below:
(1) The systematic review is relevant to vulnerable groups, defined across PROGRESS-Plus or other criteria
(2) The systematic review addresses population-level interventions and presents the findings for identifiable vulnerable groups.
(3) The systematic review includes studies targeted at the vulnerable groups.

PRISMA Checklist Item 3: Rationale
Describe the rationale for the review in the context of what is already known.

F) Equity Extension to item 3: Theory-based rationale
In the background, provide a theory-based rationale for how the intervention works, including its potential impact on health equity.

○ Strongly Agree ○ Agree ○ Disagree ○ Strongly Disagree ○ Don't Know

Comments or examples of reviews

Example: *Interventions based on the Theory of Mind cognitive model for autism spectrum disorder (ASD)* “A ’Theory of Mind intervention’ is a treatment or therapy which is explicitly or implicitly based on the Theory of Mind (ToM) cognitive model of ASD. ToM interventions target those skills which are either potential components or precursors of ToM (Swettenham2000).One example of an intervention targeting such skills is using ‘thought-bubbles’ to teach children with ASD to understand others’ thoughts and beliefs by illustrating these in bubbles (as in a cartoon) (Parsons 1999). Likewise, interventions targeting a range of social behaviours grouped together, such as general ’social skills’ training, may also be described as targeting ToM. Specific precursor skills can also be taught, such as helping a child to make eye-contact to accompany pointing to an object of interest (joint attention).”

INTRODUCTION

Reminder: Definition of equity-oriented systematic review: A review that meets one or more of the criteria below:
(1) The systematic review is relevant to vulnerable groups, defined across PROGRESS-Plus or other criteria
(2) The systematic review addresses population-level interventions and presents the findings for identifiable vulnerable groups.
(3) The systematic review includes studies targeted at the vulnerable groups.

PRISMA Checklist Item 4: Objective
Provide an explicit statement of questions being addressed with reference to participants, interventions, comparisons, outcomes, and study design (PICOS).

G) Equity Extension to item 4: Describe assessment of vulnerability
If vulnerability is used as a criteria in the review (e.g. for selecting studies, conducting analyses or judging applicability), describe how vulnerability is planned to be assessed in the methods.

○ Strongly Agree ○ Agree ○ Disagree ○ Strongly Disagree ○ Don't Know

Comments or examples of reviews

Example: *Culturally appropriate health education for type 2 diabetes mellitus in ethnic minority groups* “Participants belonged to ethnic minority communities resident in upper-middle and high-income countries. Ethnic minority communities refers to upper middle and high income countries, where there are sizeable numbers of people resident who originate from other countries, with identifiable differences in culture, religion or language, from the majority (or dominant) population and likely to be in health disadvantage.”

METHODS

Reminder: Definition of equity-oriented systematic review: A review that meets one or more of the criteria below:
(1) The systematic review is relevant to vulnerable groups, defined across PROGRESS-Plus or other criteria
(2) The systematic review addresses population-level interventions and presents the findings for identifiable vulnerable groups.
(3) The systematic review includes studies targeted at the vulnerable groups.

PRISMA Checklist Item 4: Objective
Provide an explicit statement of questions being addressed with reference to participants, interventions, comparisons, outcomes, and study design (PICOS).

H) Equity Extension to item 4: Likelihood of differences in effects

In the methods, state whether there are likely to be differences in effects between different populations that may be considered vulnerable (e.g. across PROGRESS-Plus or other criteria).

- Strongly Agree
- Agree
- Disagree
- Strongly Disagree
- Don't Know

Comments or examples of reviews

Example: *Home safety education and provision of safety equipment for injury prevention* "The socio-economic and demographic characteristics of interest were those previously found to be associated with an increased risk of childhood injury. These included child age, gender, ethnic group, family type (single or two parent), housing tenure, parental unemployment, residence in a deprived area, income, receipt of means-tested benefits, maternal education, maternal age, family size and overcrowding."

METHODS

PRISMA Checklist Item 5: Protocol and registration

Indicate if a review protocol exists, if and where it can be accessed (e.g., Web address), and, if available, provide registration information including registration number.

No equity extension proposed.

No example.

METHODS

Reminder: Definition of equity-oriented systematic review: A review that meets one or more of the criteria below:
(1) The systematic review is relevant to vulnerable groups, defined across PROGRESS-Plus or other criteria
(2) The systematic review addresses population-level interventions and presents the findings for identifiable vulnerable groups.
(3) The systematic review includes studies targeted at the vulnerable groups.

PRISMA Checklist Item 6 : Eligibility Criteria

Specify study characteristics (e.g., PICOS, length of follow-up) and report characteristics (e.g., years considered, language, publication status) used as criteria for eligibility, giving rationale.

I) Equity Extension to item 6: Rationale for study designs

In the methods, describe the type of evidence on health equity expected from the different study designs that will be included.

- Strongly Agree
- Agree
- Disagree
- Strongly Disagree
- Don't Know

Comments or examples of reviews

Examples: *Interventions to promote social cohesion in sub-Saharan Africa* "To determine study inclusion, we created a modified version of the Maryland Scientific Methods Scale (MSMS), which has been recommended for systematic reviews. We assigned a rating of one to five based on design robustness. Following the MSMS, we took a middle-ground approach and included studies that earned a rating of three or higher in our review. That is, we included studies with randomised treatment assignment or clear, quasi-experimental delineation of treatment and control groups, as well as pre-intervention and post-intervention measurement."

METHODS

Reminder: Definition of equity-oriented systematic review: A review that meets one or more of the criteria below:
(1) The systematic review is relevant to vulnerable groups, defined across PROGRESS-Plus or other criteria
(2) The systematic review addresses population-level interventions and presents the findings for identifiable vulnerable groups.
(3) The systematic review includes studies targeted at the vulnerable groups.

PRISMA Checklist Item 6 : Eligibility Criteria
Specify study characteristics (e.g., PICOS, length of follow-up) and report characteristics (e.g., years considered, language, publication status) used as criteria for eligibility, giving rationale.

J) Equity Extension to item 6: Methods of assessing differences
In the methods, state the relevance and rationale for the methods of assessing differences in outcomes between disadvantaged groups.

○ Strongly agree ○ Agree ○ Disagree ○ Strongly Disagree ○ Don't Know

Comments or examples of reviews

Example: *School feeding for improving the physical and psychosocial health of disadvantaged students* "In a meta-regression of the three RCTs (Du 2004;Neumann 2003; Powell 1998 (A)) with a total N of 1462, no significant treatment by age interaction for weight was found (Z (Q) Fixed and mixed effects = -4.58, P = 0.67). In a meta-regression of the 3 CBAs (Agarwal 1989; Bailey 1962; Devadas1979: 5-6) with a total N of 1022, there was a significant age-by-treatment interaction. The greatest benefit of school meals was shown for 5-6 year olds and 9 to 10 year olds (.95 and .89 kg respectively). The effect for 6 to 8 year old children was also large at .67 kg, but there was no effect in the Bailey study which included children up to age 13 in the analyses."

METHODS

PRISMA Checklist Item 7: Information sources
Describe all information sources (e.g., databases with dates of coverage, contact with study authors to identify additional studies) in the search and date last searched.

No equity extension item proposed.

No example.

METHODS

Reminder: Definition of equity-oriented systematic review: A review that meets one or more of the criteria below:
(1) The systematic review is relevant to vulnerable groups, defined across PROGRESS-Plus or other criteria
(2) The systematic review addresses population-level interventions and presents the findings for identifiable vulnerable groups.
(3) The systematic review includes studies targeted at the vulnerable groups.

PRISMA Checklist Item 8: Search

Present full electronic search strategy for at least one database, including any limits used, such that it could be repeated.

K) Equity Extension to item 8: Equity search strategies
In the methods, describe search strategies used to identify studies which include vulnerable populations.

Strongly Agree | Agree | Disagree | Strongly Disagree | Don't Know

Comments or examples of reviews

Example:*Effectiveness and sustainability of water, sanitation, and hygiene interventions in combating diarrhea* "Relevant studies were identified by searching academic databases pairing the following terms: 'sanitation', 'water quality', 'water quantity' or 'hygiene' against 'diarrhoea' or 'diarrhea'; and 'sanitation', 'drinking-water', or 'hygiene' against 'intervention' or 'evaluation', following Fewtrell et al. (2005). Databases of published and unpublished literature were searched using these terms, including PubMed, Embase, LILACs, Web of Science, in addition to JOLIS, IDEAS, the British Library for Development Studies (BLDS), and the Cochrane Library. Google Scholar, which has the advantage of covering all disciplines and unpublished material, was also searched, using the same search terms as above. We also personally contacted researchers working in WSH and key international organisations. Finally, we conducted bibliographic back-referencing of papers identified for inclusion and a hand-search of journals and relevant book shelves of the library of the University of Birmingham, UK. No limitations were placed on language of publication."

METHODS

PRISMA Checklist Item 9: Study selection

State the process for selecting studies (i.e., screening, eligibility, included in systematic review, and, if applicable, included in the meta-analysis).

No equity extension proposed.

No example.

PRISMA Checklist Item 10: Data collection process

Describe method of data extraction from reports (e.g., piloted forms, independently, in duplicate) and any processes for obtaining and confirming data from investigators.

No equity extension proposed.

No example.

PRISMA Checklist Item 11: Data items

List and define all variables for which data were sought (e.g., PICOS, funding sources) and any assumptions and simplifications made.

No equity extension proposed.

No example.

METHODS

PRISMA Checklist Item 12: Risk of bias in individual studies

Describe methods used for assessing risk of bias of individual studies (including specification of whether this was done at the study or outcome level), and how this information is to be used in any data synthesis.

No equity extension proposed.

No example.

PRISMA Checklist Item 13: Summary measures
State the principal summary measures (e.g., risk ratio, difference in means).

No equity extension proposed.

No example.

PRISMA Checklist Item 14: Synthesis of results
Describe the methods of handling data and combining results of studies, if done, including measures of consistency (e.g., I2) for each meta-analysis.

No equity extension proposed.

No example.

PRISMA Checklist Item 15: Risk of bias across studies
Specify any assessment of risk of bias that may affect the cumulative evidence (e.g., publication bias, selective reporting within studies).

No equity extension proposed.

No example.

METHODS

Reminder: Definition of equity-oriented systematic review: A review that meets one or more of the criteria below:
(1) The systematic review is relevant to vulnerable groups, defined across PROGRESS-Plus or other criteria
(2) The systematic review addresses population-level interventions and presents the findings for identifiable vulnerable groups.
(3) The systematic review includes studies targeted at the vulnerable groups.

PRISMA Checklist Item 16: Additional Analyses
Describe methods of additional analyses (e.g., sensitivity or subgroup analyses, meta-regression), if done, indicating which were pre-specified.

L) Equity Extension for item 16: Subgroup analysis
If a meta-analysis is planned, describe whether a subgroup analysis to explore differences between vulnerable populations was planned (defined across one or more of the PROGRESS-Plus or other criteria).

○ Strongly Agree ○ Agree ○ Disagree ○ Strongly Disagree ○ Don't Know

Comments or examples of reviews

Example: *Culturally appropriate health education for type 2 diabetes mellitus in ethnic minority groups* “The following subgroup analyses were planned: (1) we anticipated the need to stratify participants in age groups, as it can be an important effect modifier of outcomes; the effect of gender of participants, matched with gender of educators, were also analysed to assess any differences; (2) we also planned to analyse subgroups of newly diagnosed (in the first year of diagnosis), established type 2 diabetes mellitus and patients already suffering from diabetes complications; (3) we analysed subgroups of different types of health education interventions, the setting where the intervention took place (community or hospital based interventions); (4) we tried to explore difference between different literacy subgroups, ability to speak language of the majority population and countries where the interventions take place; (5) we also stratified participants in ethnic groups to identify any difference, if exists, between different ethnic groups.”

METHODS

Reminder: Definition of equity-oriented systematic review: A review that meets one or more of the criteria below:
(1) The systematic review is relevant to vulnerable groups, defined across PROGRESS-Plus or other criteria
(2) The systematic review addresses population-level interventions and presents the findings for identifiable vulnerable groups.
(3) The systematic review includes studies targeted at the vulnerable groups.

PRISMA Checklist Item 16: Additional Analyses
Describe methods of additional analyses (e.g., sensitivity or subgroup analyses, meta-regression), if done, indicating which were pre-specified.

M) Equity Extension for item 16: Context and implementation factors
In the methods, describe whether and how context and implementation factors that modify the intervention effects on health equity were assessed.

○ Strongly Agree ○ Agree ○ Disagree ○ Strongly Disagree ○ Don't Know

Comments or examples of reviews

Examples: *School feeding for improving the physical and psychosocial health of disadvantaged students* "We carefully examined several of the process elements listed above: high/low energy, compliance, substitution, and duration of the intervention. Study quality may also impact on findings; studies of lower quality often show higher effect sizes than those of higher quality...To better understand the influence of potential effect modifiers, we tabulated effects for each study sorting them by type of study, blinding versus unclear blinding, date of study, and high versus low energy."

School-Based Interventions for Aggressive and Disruptive Behavior. Update of a Meta-Analysis "Moderator analyses were then performed to identify the characteristics of the most effective programs using weighted mixed effects multiple regression with the aggressive/disruptive behavior effect size as the dependent variable. In the first stage of this analysis, the influence of study methods on effect sizes was examined. Influential method variables were carried forward as control variables for the next stage of analysis, which examined the relationships between program and student characteristics and effect size."

RESULTS

PRISMA Checklist Item 17: Study selection
Give numbers of studies screened, assessed for eligibility, and included in the review, with reasons for exclusions at each stage, ideally with a flow diagram.

No equity extension item proposed.

No Example.

RESULTS

Reminder: Definition of equity-oriented systematic review: A review that meets one or more of the criteria below:
(1) The systematic review is relevant to vulnerable groups, defined across PROGRESS-Plus or other criteria
(2) The systematic review addresses population-level interventions and presents the findings for identifiable vulnerable groups.
(3) The systematic review includes studies targeted at the vulnerable groups.

PRISMA Checklist Item 18: Study characteristics
For each study, present characteristics for which data were extracted (e.g., study size, PICOS, follow-up period) and provide the citations.

N) Equity Extension to item 18: Population description

In the results, describe the studies, including information on population characteristics and settings across the relevant PROGRESS-Plus factors or other factors.

○ Strongly Agree ○ Agree ○ Disagree ○ Strongly Disagree ○ Don't Know

Comments or examples of reviews

Example: *Lay health workers in primary and community health care for maternal and child health and the management of infectious diseases* "Of the 82 studies included in this review, 55 studies (67%) were conducted in six high income countries: Australia, Canada, Ireland, New Zealand, the UK, and the USA. Forty-one of the 82 studies were conducted in the USA. Twelve studies (14.6%) were conducted in eightmiddle income countries (Brazil, China, India, Mexico, Philipines, Thailand, Turkey, and South Africa). Fifteen trials (18.3%) were from 10 low income countries Bangladesh, Burkina Faso, Ethiopia, Ghana, Iraq, Jamaica, Nepal, Pakistan, Tanzania, and Vietnam)." "In 59 studies the intervention was delivered to patients based in their homes. Five interventions were based solely in a primary care facility (Chaisson 2001; Caulfield 1998; Merewood 2006; Olds 2002; Zaman 2008). A further eight studies involved a combination of home, primary care, and community-based interventions. Four studies delivered the intervention mainly by telephone (Dennis 2002; Dennis 2009; Graffy 2004; Singer 1999), while one implemented the intervention through community meetings (Manandhar 2004). For five studies, other sites were used such as the workplace, churches, or homeless shelters."

RESULTS

PRISMA Checklist Item 19: Risk of bias within studies
Present data on risk of bias of each study and, if available, any outcome-level assessment (see Item 12).

No equity extension item proposed.

No Example.

PRISMA Checklist Item 20: Results of individual studies
For all outcomes considered (benefits or harms), present, for each study: (a) simple summary data for each intervention group and (b) effect estimates and confidence intervals, ideally with a forest plot.

No equity extension item proposed.

No Example.

PRISMA Checklist Item 21: Synthesis of results
Present results of each meta-analysis done, including confidence intervals and measures of consistency.

No equity extension item proposed.

No Example.

PRISMA Checklist Item 22: Risk of bias across included studies
Present results of any assessment of risk of bias across studies (see Item 15).

No equity extension item proposed.

No Example.

RESULTS

Equity-extension of PRISMA

Reminder: Definition of equity-oriented systematic review: A review that meets one or more of the criteria below:
(1) The systematic review is relevant to vulnerable groups, defined across PROGRESS-Plus or other criteria
(2) The systematic review addresses population-level interventions and presents the findings for identifiable vulnerable groups.
(3) The systematic review includes studies targeted at the vulnerable groups.

PRISMA Checklist Item 23: Additional Analyses
Give results of additional analyses, if done (e.g., sensitivity or subgroup analyses, meta-regression [see Item 16]).

O) Equity Extension for item 23: Results of factors that modify the intervention In the results section, present results of factors that modify the intervention effects to assess effects on health equity (results of Q12).

○ Strongly Agree ○ Agree ○ Disagree ○ Strongly Disagree ○ Don't Know

Comments or examples of reviews

Examples: *School feeding for improving the physical and psychosocial health of disadvantaged students* “Compliance was very poor in some studies from higher income countries. For example, in the Lieberman study, only 10%of children attended breakfast 90% of the time, and less than half attended 55% or more of the time (Lieberman 1976). In this study, non-significant results were found on cognitive tests. The Tisdall study (Tisdall 1951) sheds important light on the issue as they compared ’good attenders ’to ’poor attenders’ (nearly 2/3 of the school lunch groups) in many analyses. In these analyses, the ’good attenders’ did better than the ’poor attenders’.”

School-Based Interventions for Aggressive and Disruptive Behavior. Update of a Meta-Analysis “Only two student variables were significantly associated with effect size—age and socioeconomic status. Younger students showed larger effects from universal programming than older students, and children with low socioeconomic status showed larger effects than their middle-class peers.”

DISCUSSION

PRISMA Checklist Item 24: Summary of evidence
Summarize the main findings including the strength of evidence for each main outcome; consider their relevance to key groups (e.g., health care providers, users, and policy makers).

No equity extension item proposed.

No example.

PRISMA Checklist Item 25: Limitations
Discuss limitations at study and outcome level (e.g., risk of bias), and at review level (e.g., incomplete retrieval of identified research, reporting bias).

No equity extension item proposed.

No example.

DISCUSSION

Reminder: Definition of equity-oriented systematic review: A review that meets one or more of the criteria below:
(1) The systematic review is relevant to vulnerable groups, defined across PROGRESS-Plus or other criteria
(2) The systematic review addresses population-level interventions and presents the findings for identifiable vulnerable groups.
(3) The systematic review includes studies targeted at the vulnerable groups.

PRISMA Checklist Item 26: Conclusions
Provide a general interpretation of the results in the context of other evidence, and implications for future research.

P) Equity Extension to item 26: Applicability to disadvantaged populations and settings In the results, summarize findings in terms of their applicability to the vulnerable population and/or setting of interest for this review, taking into account relevant factors (e.g. baseline risk, context, and how the intervention was implemented).

○ Strongly Agree ○ Agree ○ Disagree ○ Strongly Disagree ○ Don't Know

Comments or examples of reviews

Example: *Specialist outreach clinics in primary care and rural hospital settings* "Rural communities possibly have the most to gain from outreach in terms of client outcomes. The fact that specialist services are usually disproportionately concentrated in major urban centres results in access inequities and can compound the fact that, in resource-rich and resource-poor countries alike, rural populations are usually less healthy. We defined rural non-disadvantaged populations as those where patients must travel to another population centre for specialist or hospital care, but where access barriers are not so great that many patients would forego that care. Howe was the only Included study in the systematic review from this category. By finding that 8% more breast cancer patients received an oncology consultation and 7%more received guideline-consistent care, Howe provided an indication that access to specialist care was improved."

FUNDING

PRISMA Checklist Item 27: Funding
Describe sources of funding for the systematic review and other support (e.g., supply of data); role of funders for the systematic review.

No equity extension item proposed.

No example.

No relevant PRISMA checklist item.

Q) Are there other issues which authors need to address in equity-oriented systematic reviews, not covered in the previous list of questions? E.g. particular issues affecting specific populations, or additional items that need to be modified or added

No example.

Please tell us a little bit about yourself:

How would you classify your background and expertise (choose one)

- Clinician/practitioner
- Clinical epidemiologist
- Consumer/patient
- Methodologist
- Statistician
- Economist
- Policy-maker/manager
- Sociologist/psychologist
- Journal editor
- Systematic review author
- Funder of systematic reviews
- Other (please specify)

What is your career experience in systematic review research

- Student (BSc, MSc)
- Post-doc or PhD
- < 5 years experience
- 5-10 years experience
- > 10 years experience

Equity-extension of PRISMA

With which content area would you most associate yourself?

- Clinical health research
- Public health and health promotion
- Health systems and organization of care
- Communication
- Education
- Social Welfare
- Crime and Justice
- International development
- Other (please specify)

Are you a member of Cochrane or Campbell review group, field or methods group?

- Yes
- No

How did you hear about the survey?

- A friend or colleague asked me
- Equidad listserve
- Evidence based medicine listserve
- 3ie social network
- Twitter
- Facebook
- Cochrane blog
- BMJ blog
- Plos medicine blog
- Other (please specify)

Your name (optional)
