# Supplementary material for: PRISMA-Equity 2012 Extension: Reporting Guidelines for Systematic Reviews with a Focus on Health Equity
Source: PLoS Med. 2012 Oct 30;9(10):e1001333. doi: 10.1371/journal.pmed.1001333 (PMC3484052; doi:10.1371/journal.pmed.1001333)
Supplement: Table S3 — Distribution of online survey. (DOCX) [file pmed.1001333.s004.docx]

**Webtable S3: Distribution of online survey**

- Plos Medicine blog
- BMJ blog
- Equity listserv
- HIFA2015 listserv
- Evidence Based Health listserv
- KT Canada listserv
- CHAIN listserv
- EQUIDAD listserv
- SR Round Table listserv
- DialoguePH listserv
- Equity.cochrane.org website
- CGH website
- 3ie website
- Campbell Collaboration website
- NCCMT website
- <http://www.h2mw.eu/redactionmedicale/> website
- NCCDH website and twitter
- Cochrane facebook
