# Supplementary material for: PRISMA-Equity 2012 Extension: Reporting Guidelines for Systematic Reviews with a Focus on Health Equity
Source: PLoS Med. 2012 Oct 30;9(10):e1001333. doi: 10.1371/journal.pmed.1001333 (PMC3484052; doi:10.1371/journal.pmed.1001333)
Supplement: Table S4 — Characteristics of respondents of the survey ( n = 423). (DOCX) [file pmed.1001333.s005.docx]

**Webtable S4: Characteristics of respondents of the survey (n=423)**

|  |  | Respondents  N (%) |
| --- | --- | --- |
| Background and Expertise |  |  |
|  | Systematic review author | 83 (25.9) |
|  | Clinician/practitioner | 50 (15.6) |
|  | Methodologist | 36 (11.2) |
|  | Epidemiologist | 28 (8.7) |
|  | Policy maker/manager | 28 (8.7) |
|  | Sociologist/psychologist | 23 (7.2) |
|  | Journal editor | 8 (2.5) |
|  | Economist | 6 (1.9) |
|  | Patient | 6 (1.9) |
|  | Funder of systematic reviews | 4 (1.2) |
|  | Statistician | 4 (1.2) |
|  | Other | 45 (14.0) |
| Career experience in systematic reviews |  |  |
|  | >10 years experience | 85 (27.2) |
|  | 5-10 years experience | 73 (23.4) |
|  | <5 years experience | 82 (26.3) |
|  | Post-doc or PhD | 45 (14.4) |
|  | Student (BSc, MSc) | 27 (8.7) |
| Member of Cochrane or Campbell review group, field, or methods group |  |  |
|  | Yes | 140 (44.2) |
|  | No | 177 (55.8) |
| Heard about the survey through |  |  |
|  | Colleague | 85 (26.7) |
|  | Evidence based medicine listserv | 65 (20.4) |
|  | EQUIDAD listserv | 49 (15.4) |
|  | Cochrane blog | 9 (2.8) |
|  | BMJ blog | 7 (2.2) |
|  | PLoS medicine blog | 3 (0.9) |
|  | Twitter | 3 (0.9) |
|  | Facebook | 2 (0.6) |
|  | 3ie social network | 2 (0.6) |
|  | Other | 93 (29.2) |
