# Supplementary material for: PRISMA-Equity 2012 Extension: Reporting Guidelines for Systematic Reviews with a Focus on Health Equity
Source: PLoS Med. 2012 Oct 30;9(10):e1001333. doi: 10.1371/journal.pmed.1001333 (PMC3484052; doi:10.1371/journal.pmed.1001333)
Supplement: Table S5 — Responses to online survey. (DOCX) [file pmed.1001333.s006.docx]

**Webtable S5: Responses to online survey**

| **Proposed equity extension item** | **Responses (%)** | | | | |
| --- | --- | --- | --- | --- | --- |
|  | Strongly Agree | Agree | Disagree | Strongly disagree | Don’t Know |
| **Reporting health equity is important in systematic reviews.** | 61.7 | 33.3 | 2.4 | 1.2 | 1.4 |
| **1 A: Equity in title** | 37.3 | 47.9 | 9.3 | 1.0 | 4.5 |
| **2A: Abstract reports how health equity is considered** | 51.8 | 39.3 | 5.5 | 1.3 | 2.1 |
| **2B: Abstract report broad search strategy related to equity questions** | Not assessed | | | | |
| **2C: Abstract reports effects on health equity** | 48.5 | 40.4 | 7.0 | 0.8 | 3.3 |
| **2D: Abstract describes limits of applicability to disadvantaged populations** | Not assessed | | | | |
| **3B: Analytic framework shows effects on health equity** | 31.4 | 42.8 | 14.7 | 2.8 | 8.3 |
| **3A: Rationale related to health inequity** | 33.1 | 47.3 | 12.6 | 1.7 | 5.3 |
| **4A: Describe how disadvantage is planned to be assessed** | 48.0 | 41.8 | 5.1 | 0.9 | 4.3 |
| **4B: Likelihood of different relative effects for any of PROGRESS+ populations** | 38.8 | 46.3 | 8.6 | 1.1 | 5.2 |
| **6A: Rationale for eligible study designs to assess effects on health inequalities** | 33.8 | 44.5 | 13.6 | 1.5 | 6.5 |
| **6B: Outcomes relevant for disadvantaged** | 36.3 | 51.4 | 6.6 | 0.6 | 5.1 |
| **7A and 8A: Equity search strategies** | 46.2 | 39.3 | 9.6 | 1.5 | 3.3 |
| **11A- Data items related to equity** | Not assessed | | | | |
| **14A: Methods of synthesizing health inequalities** | Not assessed | | | | |
| **16A: Subgroup analysis related to health equity** | 47.9 | 43.0 | 4.8 | 1.5 | 2.7 |
| **16A: Effect Modifiers related to health equity** | 43.0 | 47.0 | 4.9 | 0.9 | 4.3 |
| **18A: Population description: Characteristics of disadvantage** | 51.8 | 40.9 | 5.2 | 0.3 | 1.8 |
| **21A: Results of effect modifiers related to equity** | 46.9 | 44.2 | 4.3 | 0.6 | 4.0 |
| **23A: Results of additional synthesis approaches related to equity** | Not assessed | | | | |
| **26A: Applicability to disadvantaged populations and settings** | 57.2 | 37.5 | 2.8 | 0.6 | 1.8 |
| **26B. Provide implications for research, practice or policy related to health equity (e.g. types of research needed to address unanswered questions).** | Not assessed |  |  |  |  |
