# Supplementary material for: PRISMA-Equity 2012 Extension: Reporting Guidelines for Systematic Reviews with a Focus on Health Equity
Source: PLoS Med. 2012 Oct 30;9(10):e1001333. doi: 10.1371/journal.pmed.1001333 (PMC3484052; doi:10.1371/journal.pmed.1001333)
Supplement: Table S6 — Consensus meeting agenda. (DOCX) [file pmed.1001333.s007.docx]

**Webtable S6: Consensus Meeting Agenda**

**Equity-extension of PRISMA CONSENSUS MEETING AGENDA: February 9-10, 2012**

**Sfondrata, Bellagio, Rockefeller Centre**

**Wed Feb 8, 2012**

**7-8:30 pm Registration, reception and dinner**

**Thursday Feb 9, 2012**

|  |  | ***Coleads*** | ***Discussant*** | ***Topic*** |
| --- | --- | --- | --- | --- |
| 0800 – 0900 |  |  |  | *Breakfast* |
| **Morning session** | **Chair:** David Tovey |  |  |  |
| 0900 – 0945 |  | Mark Petticrew, Peter Tugwell, Vivian Welch |  | Welcome, introductions  Overview of goals and process |
| 0945 – 1010 |  | Mark Petticrew | Liz Wager | Background: Reporting Guidelines |
| 1010- 1050 |  | Peter Tugwell |  | **Focus of checklist:** Why equity and systematic reviews, when is it important, what types of questions and reviews? Who will use this extension checklist? |
| 1050 – 1105 |  |  |  | *Break* |
| 1105 – 1130 |  | Vivian Welch |  | What do we know about equity and reporting in SRs?  Survey and Results |
| 1130 – 1145 |  | Peter Tugwell | Prathap Tharyan | Equity Extension A: **Title and equity** |
| 1145 – 1200 |  | Mark Petticrew | Prathap Tharyan | Equity Extension B and C: **Abstract and equity-methods; Abstract and equity-effects** |
| 1200 – 1230 |  | Vivian Welch | Liz Waters, Tracey Koehlmoos-Perez | Equity Extension D: **Logic model/analytic framework and equity** |
| 1230 - 1300 |  | Howard White | Liz Waters, Tracey Koehlmoos-Perez | Equity Extension E: **Rationale for equity effects** |
| 1300 – 1400 |  |  |  | *Lunch* |
| **Afternoon session** | **Chair: Trish Groves** |  |  |  |
| 1400 – 1430 |  | Vivian Welch | Shally Awasthi | Equity Extension F: **Defining disadvantage** |
| 1430 – 1445 |  | Andy Oxman | Kent Ranson | Equity Extension G: **Likelihood of different relative effects and equity** |
| 1445 – 1515 |  | Mark Petticrew, Terri Pigott | Tomas Pantoja | Equity Extension H: **Study design to consider equity** |
| 1515 – 1530 |  |  |  | *Break* |
| 1530 – 1600 |  | Peter Tugwell |  | Equity Extension I: **Relevance of outcomes for disadvantaged** |
| 1600 – 1630 |  | Mark Petticrew | Luis Gabriel Cuervo | Equity Extension J: **Searching for equity** |
| 1630 – 1700 |  | Mark Petticrew, Peter Tugwell, Vivian Welch |  | Wrap-up, tabled issues |
| 1900 – 2030 |  |  |  | Dinner |

**Friday Feb 10, 2012**

|  |  | ***Coleads*** | ***Discussant*** | ***Topic*** |
| --- | --- | --- | --- | --- |
| 0800 – 0830 |  |  |  | *Breakfast* |
| **Morning session** | **Chair:** Virginia Barbour |  |  |  |
| 0830 – 0845 |  | Mark Petticrew, Peter Tugwell, Vivian Welch |  | Summary of Day 1 |
| 0845 – 0915 |  | Vivian Welch | Kent Ranson | Equity Extension K: **Measures to assess effects on health inequalities** |
| 0915 – 0945 |  | Vivian Welch | Shally Awasthi | Equity Extension L: **Describing population characteristics of disadvantage** |
| 0945 – 1015 |  | Howard White | Tessa TanTorres | Equity Extension M: **Planned effect modifiers and equity** |
| 1015 – 1030 |  |  |  | *Coffee Break* |
| 1030 – 1100 |  | Mark Petticrew, Terri Pigott | Zulfiqar Bhutta | Equity Extension N: **Subgroup analysis to consider equity** |
| 1100 – 1130 |  | Howard White | Zulfiqar Bhutta | Equity Extension O: **Results of effect modifiers and equity** |
| 1130 – 1200 |  | Andy Oxman | Tomas Pantoja | Equity Extension P: **Applicability and equity** |
| 1200 – 1230 |  | Mark Petticrew, Peter Tugwell, Vivian Welch |  | Post meeting activities: writing committee, drafting, publication, authorship, research agenda |
| 1230 – 1300 |  | Trish Groves, Virginia Barbour | David Tovey & Liz Wager | *Presentation*: Other KT activities  Implementing PRISMA-Equity: how to involve funding agencies, journals, and other potential users |
| 1300 – 1400 |  |  |  | *Lunch* |
| **Afternoon session** | **Chair**: Zulfiqar Bhutta |  |  |  |
| 1400 – 1500 |  | Trish Groves, Virginia Barbour | David Tovey & Liz Wager | *Discussion*: Other KT activities  Implementing PRISMA-Equity: how to involve funding agencies, journals, and other potential users |
| 1500 – 1530 |  | Mark Petticrew, Peter Tugwell, Vivian Welch |  | wrap-up |
