# Supplementary material for: PRISMA-Equity 2012 Extension: Reporting Guidelines for Systematic Reviews with a Focus on Health Equity
Source: PLoS Med. 2012 Oct 30;9(10):e1001333. doi: 10.1371/journal.pmed.1001333 (PMC3484052; doi:10.1371/journal.pmed.1001333)
Supplement: Table S7 — Participant roles. (DOCX) [file pmed.1001333.s008.docx]

**Webtable S7: Participant Roles**

**Responsibilities of the Leads**

1. Give a two minute presentation of the issues.

**Responsibilities of the Chairs**

1. Ensures that the group stays on topic.
2. Ensure that the discussion stays on time.
3. Smooth the group process to ensure that everyone's opinion is heard.
4. Table important issues so they can be addressed in a later discussion.
5. Convene a vote or measure of agreement at end of time slot. (Reminder: we are not voting on the exact wording of the items at this point).

**Responsibilities of the Discussants**

1. Read the background materials on the session.
2. Start the discussion with a one-minute reaction to the Lead’s presentation.
