# Supplementary material for: PRISMA-Equity 2012 Extension: Reporting Guidelines for Systematic Reviews with a Focus on Health Equity
Source: PLoS Med. 2012 Oct 30;9(10):e1001333. doi: 10.1371/journal.pmed.1001333 (PMC3484052; doi:10.1371/journal.pmed.1001333)
Supplement: Table S8 — Systematic reviews with equity in the title (1-08-2011 to 1-08-2012). (DOCX) [file pmed.1001333.s009.docx]

**Webtable S8: Systematic reviews with equity in the title (1-08-2011 to 1-08-2012)**

| **Query** | **Items Found** |
| --- | --- |
| **(equit*[ti] OR inequit*[ti]) AND (MEDLINE[Title/Abstract] OR (systematic[Title/Abstract] AND review[Title/Abstract]) OR meta-analysis[Publication Type])** | 7 |
| **(MEDLINE[Title/Abstract] OR (systematic[Title/Abstract] AND review[Title/Abstract]) OR meta-analysis[Publication Type])** | 14420 |
